# The mathematical expression of damage law of museum lighting on dyed artworks

**DOI:** 10.1038/s41598-021-90520-z

**Published:** 2021-05-26

**Authors:** Rui Dang, Baoping Wang, Xiangyang Song, Fenghui Zhang, Gang Liu

**Affiliations:** Tianjin Key Laboratory of Architectural Physics and Environmental Technology, School of Architecture, Tianjin University, Tianjin, 300072 China

**Keywords:** Optics and photonics, Physics

## Abstract

Dyed artworks are highly sensitive to light and are easily affected by museum lighting, resulting in irreversible permanent color damage such as fading and discoloration. Exposure, light source spectrum and material properties are the three indicators causing damage to artworks. Therefore, it is the basis for effective lighting protection to reveal the quantitative influence of exposure and light source spectrum composition on the damage degree of different pigments and establish a mathematical model that can accurately express the above rules. At present, the color damage calculation model of dyed artworks under three parameters’ coupling action is missing. This research established a visual three-dimensional change surface of the color difference values of 23 pigments varying with the spectral wavelength and exposure through experimental methods. The relative responsivity function Δ*E*_*n*_ = *f*_*n*_(*λ*, *Q*), where n = 1 ~ 23, was obtained for 23 pigments under the coupling effects of exposure and light source spectra. Furthermore, a mathematical model $D_{n} = \mathop \smallint \limits_{380}^{780} S\left( \lambda \right) \cdot f_{n} \left( {\lambda ,Q} \right)d\lambda$ calculating the color damage of pigments in the range of visible light was proposed. The proposed model was verified by the experimental method, which realizes the mathematical expression of the damage law of museum lighting on dyed artworks.

## Introduction

The light environment of museums is one of the most complex architectural environments, which needs to consider the dual needs of cultural relics protection and visitor viewing. At present, At present, there are a lot of researches on visitor viewing in the light environment of museums^1,2^. However, considering the protection of cultural relics is the basis of the museum light environment, the lighting damage to cultural relics is the focus of researches on museum lighting. Museum lighting is an important factor causing damage to cultural relics^3,4^. In particular, paintings, colored sculptures, colored silk fabrics and other dyed artworks exhibit the highest photosensitivity stipulated by the International Commission on illumination (CIE)^5,6^. Due to their material characteristics, they are easily affected by light. They are prone to irreversible, permanent color damage, such as fading, discoloration and blackening, which seriously affects the historical and artistic value of cultural relics^7^.

Previous studies have shown that the fundamental reason for the color damage of dyed cultural relics is the photochemical reaction that occurs after the illuminated pigment continuing to absorb the spectral energy of the light source^5^. Firstly, the color damage degree of pigments is related to the exposure (the product of irradiation intensity and exposure time)^8^. Secondly, due to the significant difference in the materials of different pigments and the diversity of spectral power distribution (SPD) of varying light sources, the absorption and reflection characteristics of pigments to the spectrum are different^9^. There are significant differences in the degree of damage caused by different light sources to various pigments. Therefore, exposure, the material characteristics of pigments and SPD are the three key parameters causing the damage to artworks. It is the basis of adequate lighting protection to reveal the quantitative influence of three critical parameters on the pigment color damage and establish the mathematical model that can accurately describe the law. However, the exposure is a free variable, and there are many types of pigments, and the SPD varies greatly. Therefore, the photochemical reaction process under multi-parameter combination conditions is very complicated.

To realize the mathematical expression of the damage law of museum lighting on dyed artworks, many scholars have carried out relevant researches. The light aging experiment is a popular international method to study the lighting damage of cultural relics^10,11^, and the selection of experimental evaluation parameters is the key. As a mature chromatic index, the color difference is widely used in the evaluation of color damage of dyed cultural relics^12–14^. It is a quantitative evaluation of color change by detecting specimens’ color coordinates before and after irradiation and calculating the color difference value by using the formula. Based on the color difference and light aging experiment method, scholars have made progress on the color damage law of dyed artworks.

In terms of the relationship between exposure and material damage, the Berlin model proposed that the damage to cultural relics is a function of effective radiation exposure and was expressed by a simple curve^5,15^. Obviously, considering a single curve can not express all the materials in the museum, it is necessary to study the damage of different materials in the museum. Luo obtained the color difference of photographic materials with exposure under different light sources^16^. By using data fitting method, Rui Dang obtained the damage function of traditional Chinese painting pigments with illuminance and irradiation time under halogen lamp^17^. Therefore, the current researches have proved that there is a clear relationship between exposure and material damage, and the damage law is different for different materials. However, the experimental light source is the traditional light source with fixed spectrum, which has some limitations. Therefore, it is necessary to discuss the damage effect of light source SPD on different materials.

In 1953, Harrison studied the relationship between SPD and material damage for the first time, and concluded that the relative damage of low-grade paper in the visible light range decreased with the increase of wavelength^18^. On this basis, by using different narrow-band lights filtered from xenon lamp to conduct the light aging experiment on five oil painting materials, Aydinli obtained the spectral response function of each material^19,20^. With the extensive use of LED light sources with flexible spectral composition in the lighting of dyed artworks^21,22^, there are a lot of researches on the response of different pigments to the LED spectrum. Some studies compared the fading effect of LED light sources with different color temperatures with that of traditional light sources, and found that white LED has lower damage to materials^23–25^. Taking yellow pigments as the research object, Lunz found that not all short wavelengths cause more damage than long wavelengths, proving that different materials have the different wavelength dependence^26^. Rui Dang used ten kinds of narrow-band LEDs to irradiate the pigment samples continuously, and obtained the response functions of different visible spectrum bands to inorganic pigments^27^. However, these studies are limited to the damage effect of light source SPD on cultural relics, without combining the coupling effect of exposure and light source SPD parameters, and lack of mathematical expression of the damage model.

In summary, the color difference and the light aging experiment are the general methods for the damage research on museum dyed artworks. The experiment of different band narrow band light is an effective method to obtain the damage law. However, there is a lack of discussion on the coupling effect of multi-parameters. Especially, the mathematical model of color damage under the coupling effect of SPD, exposure and pigments type is still not obtained. Therefore, this study aims to obtain the mathematical model calculating the color damage under the coupling effect of three parameters. In this study, 10 kinds of narrow band LEDs were used as experimental light sources, 23 kinds of art pigments were used as experimental samples, and exposure was used as experimental variable. The method of color difference was used to evaluate the lighting damage, and the mathematical models of 23 pigments under the coupling effect of light source SPD and exposure were established. Then the accuracy of models was verified by using the method of typical light source irradiating samples, so as to realize the mathematical expression of the damage law of museum lighting on dyed artworks.

## Materials and methods

### Experimental light sources

**Figure 1:**
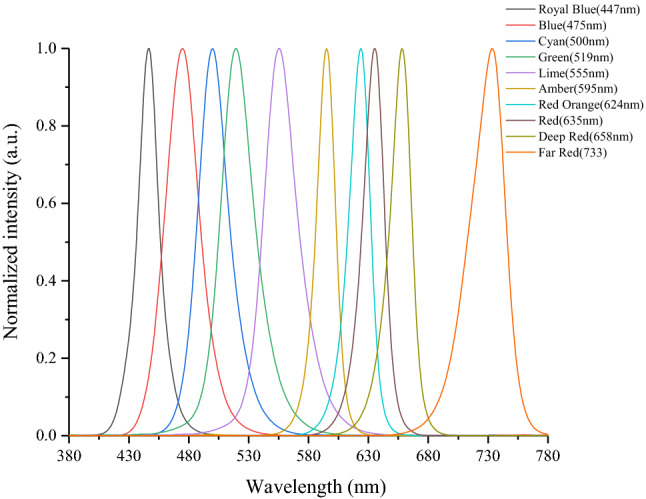
The relative SPDs of ten narrow-band light sources.

Ten kinds of narrow-band light sources with different peak wavelengths in the visible spectrum range were prepared by LUXEON C Color Line monochromatic LED chip. The relative SPDs of light sources measured by photo research PR 670 Spectroradiometer is shown in Fig. 1. At the same time, due to the long experimental time, in order to avoid the influence of light source attenuation on the experimental results, the luminous flux of the light source was measured in each measurement period, and the aging light source would be replaced by a backup light source as soon as the attenuation was detected^27,28^.

### Experimental specimens

**Figure 2:**
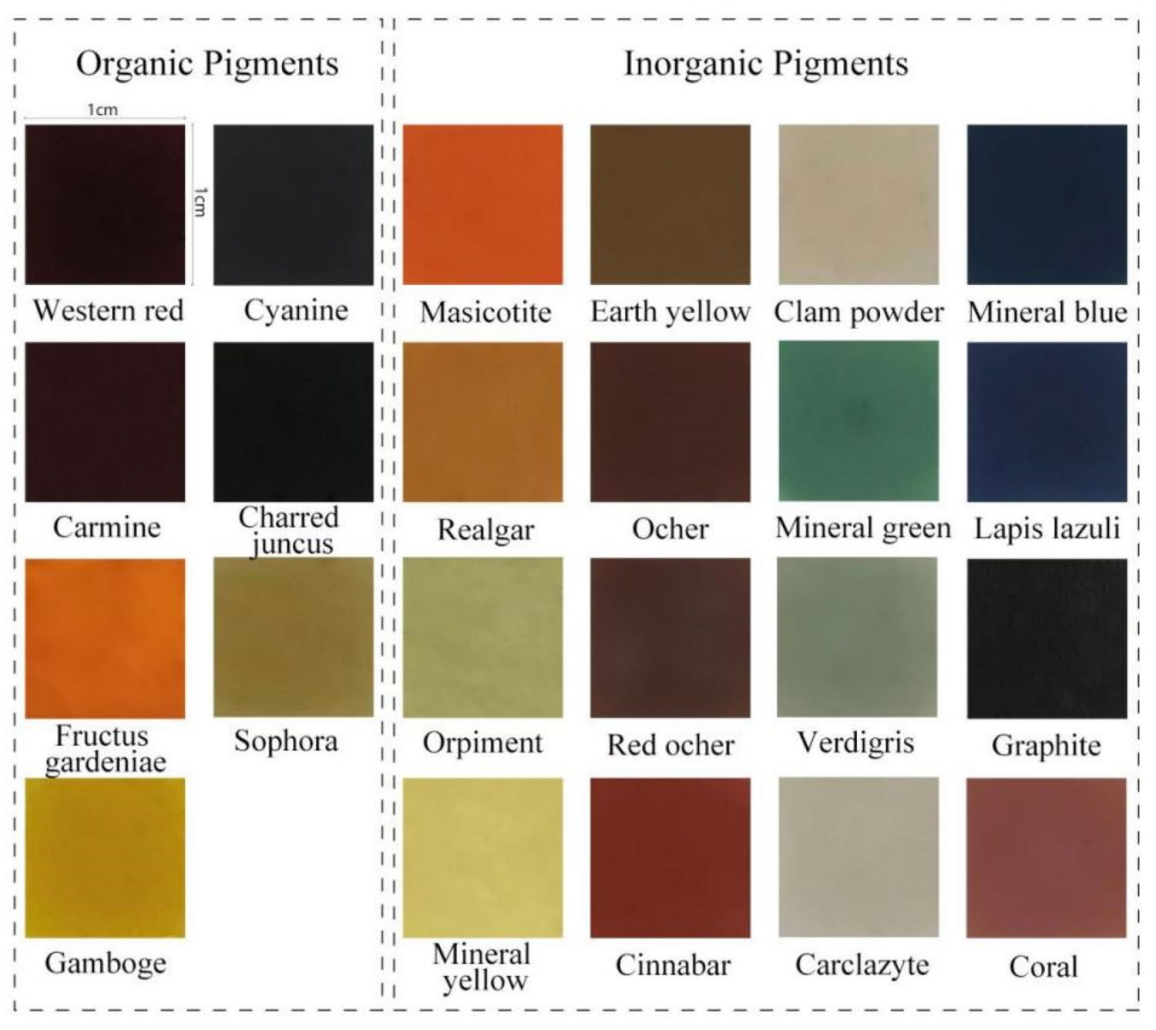
Experimental sample diagram.

A total of 23 kinds of pigments, including 16 kinds of inorganic pigments and 7 kinds of organic pigments, including almost the pigment types of common dyed artworks^29,30^. The specimen preparation method was as follows: Firstly, a 1*1 cm square hole was carved on the UV film (1.25 mm thick), and the UV film was pasted on the mounted paper substrate (watercolor paper). Secondly, the pigment was mixed with gelatin and water in a mass ratio of 1:1:10. Then, the prepared pigment solution was evenly applied in the square hole. Finally, the specimen was dried in a dark environment with an average temperature of 25℃ and a humidity of 50 ± 5% for five months to ensure the stability of the color parameter^31^. The experimental specimens were obtained by the above method (Fig. 2). On the basis of the above method, ten groups of the same specimens were made.

### Experimental program

The experiment was carried out in the underground all dark optical laboratory of Tianjin University. Set up a lighting experiment box with the function of temperature and humidity automatic adjustment, in order to keep the physical environment in the necessary conditions for the preservation of cultural relics in museums. The temperature, relative humidity and ventilation rate of the whole experiment box were 23 ± 0.5℃, 50%, and 0.5 d^−1^ respectively^31,32^.

**Figure 3:**
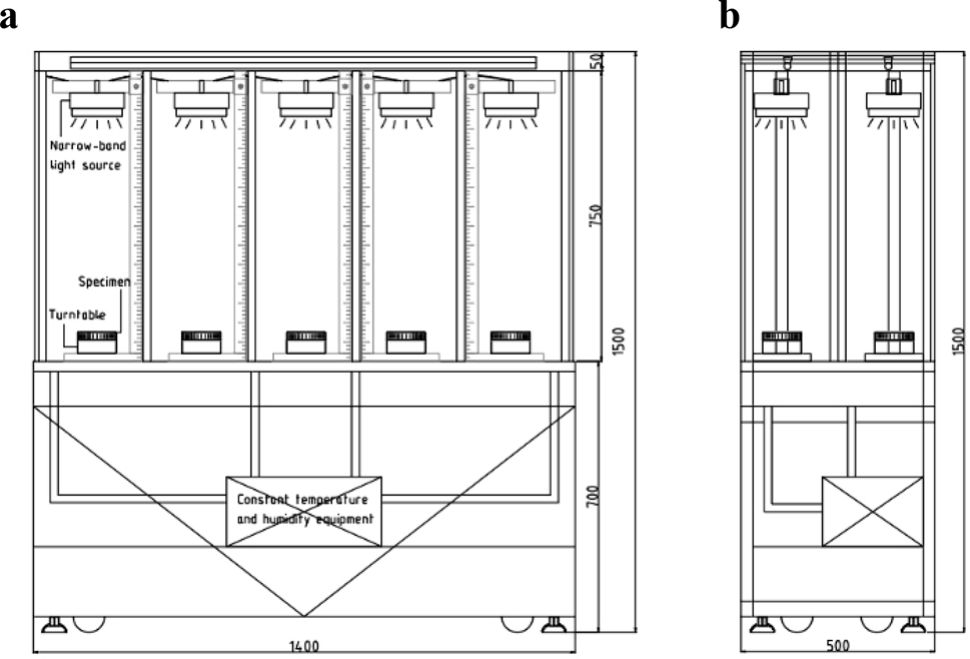
Experimental device diagram. (**a**) The front elevation, (**b**) The side elevation.

The experiment was divided into ten irradiation groups according to the types of narrow-band light sources and carried out simultaneously. Firstly, the experiment box was divided into ten independent spaces with partitions, to avoid interference from different irradiation groups. Then, ten narrow-band light sources were respectively installed in the upper part of each separate area, and ten identical specimens were placed under various light sources. Lastly, by adjusting the output power and height of the light source, the irradiance on the surface of ten specimens was fixed at 10.000 ± 3% W/m^2^. At the same time, ten automatic rotating turntables were installed on the experimental platform, to ensure the uniformity of irradiance on the surface of specimens. The experimental device is shown in Fig. 3.

The specimens were exposed periodically, and the exposure was accumulated with the experiment. The exposure was 2400 Wh/m^2^ as a measurement cycle. After each exposure cycle, the color parameters were measured. During the measurement, the specimens were taken out of the experimental box, and CIE L*a*b* color parameters of 23 specimens were measured with Topcon BM-5 color luminance meter under CIE standard A light source (thermal radiation light source with a correlated color temperature of 2680 K calibrated by the Chinese Metrology Institute). The HJS-480-0-12 voltage stabilizer was used to ensure the stability of the standard A light source's output power. At the same time, the tester wore the special black lab suit for measurement to avoid the influence of external interference on the measurement result.

## Results

### Influence of exposure and SPD on pigment color change

According to the CIE L^*^a^*^b^*^ color coordinates of 23 kinds of pigment samples under 10 kinds of narrow-band light irradiation, L_0_^*^a_0_^*^b_0_^*^ is the color coordinate of the pigment in the initial unirradiated state, and L_1_^*^a_1_^*^b_1_^*^ ~ L_6_^*^a_6_^*^b_6_^*^ is the color coordinate of the pigment measured in six test cycles, respectively. Using the Eq. (1) to calculate the color difference ΔE^*^_1_ ~ ΔE^*^_6_ of the pigment relative to the initial non-irradiated state can be calculated.

1 $$ \begin{aligned} & \Delta E^{*}_{1} = \sqrt {(L^{*}_{1} - L^{*}_{0} )^{2} + (a^{*}_{1} - a^{*}_{0} )^{2} + (b^{*}_{1} - b^{*}_{0} )^{2} } \\ & \Delta E^{*}_{2} = \sqrt {(L^{*}_{2} - L^{*}_{0} )^{2} + (a^{*}_{2} - a^{*}_{0} )^{2} + (b^{*}_{2} - b^{*}_{0} )^{2} } \\ & \cdots \cdots \\ & \Delta E^{*}_{6} = \sqrt {(L^{*}_{6} - L^{*}_{0} )^{2} + (a^{*}_{6} - a^{*}_{0} )^{2} + (b^{*}_{6} - b^{*}_{0} )^{2} } \\ \end{aligned} $$

According to the Eq. (1), the color difference change values of 23 pigments can be obtained. Taking graphite pigment as an example, under the coupling effect of SPD and exposure, the color difference of graphite pigment is shown in Table 1:

**Table 1:** The color difference values of graphite pigment in different exposure periods under irradiation of 10 narrow-band lights.

	Wavelength(nm)									
Exposure(Wh/m^2^)	447	475	500	519	555	595	624	635	658	733
0	0.000	0.000	0.000	0.000	0.000	0.000	0.000	0.000	0.000	0.000
2400	1.014	0.880	0.901	0.744	0.779	0.645	0.659	0.708	0.591	0.517
4800	1.229	1.042	1.250	1.022	1.019	0.898	0.829	0.822	0.813	0.635
7200	1.712	1.420	1.621	1.390	1.112	0.963	1.090	1.075	0.936	0.845
9600	1.891	1.710	1.886	1.674	1.308	1.394	1.174	1.136	1.080	1.029
12,000	2.071	1.923	1.954	2.027	1.715	1.571	1.311	1.529	1.182	1.166
14,400	2.447	2.043	2.267	2.129	1.914	1.648	1.762	1.652	1.349	1.212

**Figure 4:**
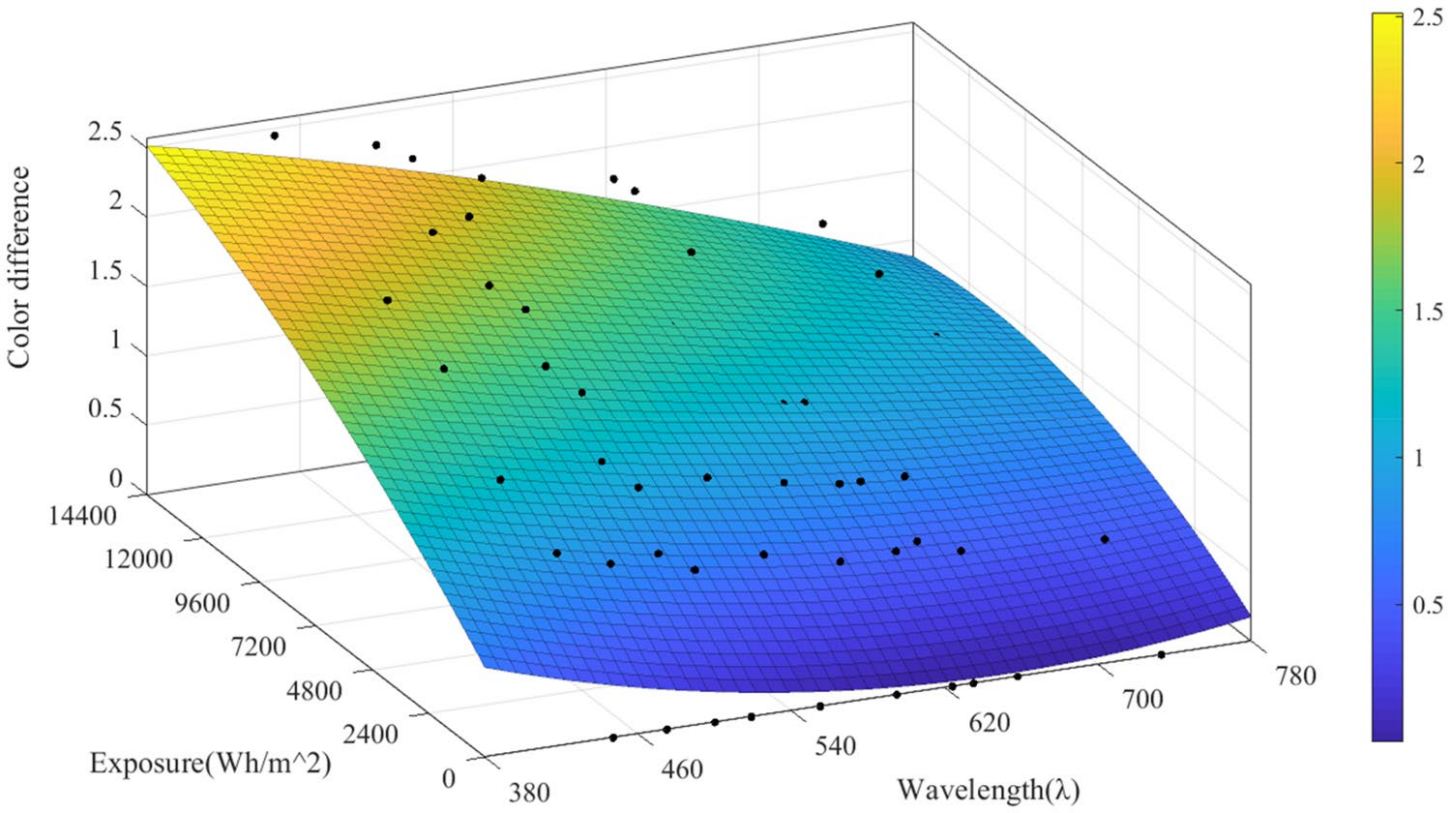
The three-dimensional variation surface of graphite’s color difference varying with SPD and exposure.

According to the peak wavelength of the narrow-band lights and exposure index adopted in the experiment, combined with the color difference calculation results, the three-dimensional variation surface of color difference of 23 kinds of pigments was obtained. It takes wavelength (*λ*) as x-axis, exposure (*Q*) as y-axis, and the color difference (ΔE*) as z-axis. It can characterize the coupling effect of SPD and exposure on the color damage of a certain pigment. Taking graphite as an example, the three-dimensional variation surface of its color difference is shown in Fig. 4. The color difference increases with the decrease of wavelength and the increase of exposure:

### The relative responsivity function of pigment color to exposure and SPD

The surface is fitted as a binary function Δ*E*_n_^*^ = *f*_n_(*λ*, *Q*), where n = 1 ~ 23, representing 1 ~ 23 pigments. This function can realize the mathematical description of the coupling effect of exposure and SPD on the color of a specific pigment, so as to quantitatively calculate the color change. Taking graphite pigment as an example, the relative responsivity function to exposure and SPD is shown in Eq. (2), and the goodness of fit of the equation is R^2^ = 0.9523.

2 $$ \begin{aligned} \Delta E_8^* & = f_8(\lambda ,Q) = 3.608 - 0.01101*\lambda + 8.478e - 06*\lambda^{2} \\ & \quad + 7.769e - 07*\lambda *Q - 1.551e - 09*Q^{2} \\ & \quad - 5.851e - 12*\lambda *Q^{2} - 7.714e - 10*\lambda^{2} *Q \\ \end{aligned} $$

According to the above method, the relative responsivity functions of all 23 pigments to exposure and SPD were obtained, as shown in Table 2.

**Table 2:** The relative responsivity functions of 23 pigments to exposure and SPD.

Organic pigments			
Serial number	Pigments	Functions	R^2^
1	Carmine	*f*_1_(*λ*,*Q*) = 0.4599 + 0.08805*λ + 0.0003333*Q − 0.0004322*λ^2 + 9.068e − 07*λ*Q − 5.31e − 08*Q^2 + 6.875e − 07*λ^3–6.282e − 10*λ^2*Q–2.921e − 11*λ*Q^2 + 2.883e − 12*Q^3–3.576e − 10*λ^4	0.8103
2	Gamboge	*f*_2_(*λ*,*Q*) = 63.42–0.3323*λ–0.0005789*Q + 0.0005715*λ^2 + 5.601e − 07*λ*Q + 9.045e − 09*Q^2–3.224e − 07*λ^3 + 1.34*λ^(− 0.4619)*Q^0.468	0.8002
3	*F*ructus gardeniae	*f*_3_(*λ*,*Q*) = − 11.95 + 0.04481*λ + 0.0007249*Q–3.9e−05*λ^2 + 3.824e−08*λ*Q–2.598e−08*Q^2	0.8297
4	Cyanine	*f*_4_(*λ*,*Q*) = 0.0119 + 0.7943*λ + 0.003569*Q-0.005318*λ^2–1.15e−05*λ*Q-2.736e−07*Q^2 + 1.321e−05*λ^3 + 9.942e−09*λ^2*Q + 9.416e−10*λ*Q^2–1.443e−08*λ^4 + 5.853e−12*λ^5–8.185e−13*λ^2*Q^2	0.8044
5	Charred juncus	*f*_5_(*λ*,*Q*) = 3.628–0.01353*λ–0.0001534*Q + 1.246e−05*λ^2 + 2.207e−06*λ*Q–2.263e−08*Q^2–2.37e−09*λ^2*Q + 2.583e−11*λ*Q^2	0.8188
6	Sophora	*f*_6_(*λ*,*Q*) = 0.7007 + 0.02494*λ + 0.005665*Q-8.825e−05*λ^2–1.132e−05*λ*Q-2.345e−07*Q^2 + 6.316e−09*λ^2*Q + 1.766e−10*λ*Q^2 + 4.758e−12*Q^3 + 7.38e−08*λ^3	0.9258
7	Western red	*f*_7_(*λ*,*Q*) = − 74.77 + 0.4194*λ + 0.002009*Q–0.0007665*λ^2–1e−06*λ*Q − 1.833e−07*Q^2 + 4.577e−07*λ^3 + 4.258e−11*λ*Q^2 + 6.081e−12*Q^3	0.8826

Inorganic pigments			
Serial number	Pigments	Functions	R^2^
8	Graphite	*f*_8_(*λ*,*Q*) = 3.608–0.01101*λ + 8.478e−06*λ^2 + 7.769e−07*λ*Q–1.551e−09*Q^2–5.851e−12*λ*Q^2–7.714e−10*λ^2*Q	0.9523
9	Masicotite	*f*_9_(*λ*,*Q*) = 310.6–2.08*λ + 0.001423*Q + 0.005159*λ^2–7.754e−08*λ*Q–1.459e−07*Q^2–5.615e−06*λ^3 + 5.353e−12*Q^3 + 2.265e−09*λ^4	0.8161
10	Cinnabar	*f*_10_(*λ*,*Q*) = − 74.12 + 0.3771*λ + 0.001191*Q–0.0006256*λ^2–1.638e−07*λ*Q–1.244e−07*Q^2 + 3.391e−07*λ^3 + 1.902e−10*λ^2*Q + 1.13e−12*λ*Q^2 + 4.576e−12*Q^3	0.8592
11	Realgar	*f*_11_(*λ*,*Q*) = 869.6–5.958*λ + 0.0009322*Q + 0.01508*λ^2 + 2.18e−07*λ*Q–1.058e−07*Q^2–1.671e−05*λ^3 + 3.784e−12*Q^3 + 6.839e−09*λ^4	0.8669
12	Coral	*f*_12_(*λ*,*Q*) = − 0.969 + 0.02554*λ + 0.0003999*Q–7.512e−05*λ^2–1.446e−07*λ*Q–3.256e−08*Q^2 + 5.765e−08*λ^3 + 1.255e−12*Q^3	0.8109
13	Ocher	*f*_13_(*λ*,*Q*) = − 424.7 + 3.015*λ + 0.0005588*Q–0.00794*λ^2–2.651e−08*λ*Q–5.834e−08*Q^2 + 9.194e−06*λ^3 + 2.248e−12*Q^3–3.949e−09*λ^4	0.8022
14	Red ocher	*f*_14_(*λ*,*Q*) = − 2.357 + 0.01163*λ + 0.0005763*Q–1.934e−05*λ^2 + 9.957e−08*λ*Q–7.012e−08*Q^2 + 1.112e−08*λ^3 + 2.661e−12*Q^3	0.8179
15	Earth yellow	*f*_15_(*λ*,*Q*) = − 2.427 + 0.009985*λ + 0.0005433*Q–8.401e−06*λ^2–2.785e−07*λ*Q–1.44e−08*Q^2	0.8081
16	Mineral yellow	*f*_16_(*λ*,*Q*) = 3.94–0.01106*λ + 0.001674*Q + 8.739e−06*λ^2–2.404e−06*λ*Q–7.75e−08*Q^2 + 5.513e−10*λ^2*Q + 9.863e−11*λ*Q^2	0.8240
17	Mineral blue	*f*_17_(*λ*,*Q*) = − 18.58 + 0.2373*λ + 0.000173*Q + -0.0008983*λ^2 + 5.137e−07*λ*Q–1.725e−08*Q^2 + 1.349e−06*λ^3–3.704e−10*λ^2*Q–1.051e−11*λ*Q^2 + 8.028e−13*Q^3–7.069e−10*λ^4	0.8119
18	Mineral yellow	*f*_18_(*λ*,*Q*) = 0.179–0.1169*λ + 0.001289*Q + 0.0006169*λ^2–5.422e−07*λ*Q–1.072e−07*Q^2–1.058e−06*λ^3 + 3.928e−12*Q^3 + 5.899e−10*λ^4	0.8269
19	Lapis lazuli	*f*_19_(*λ*,*Q*) = − 1.491 + 0.08115*λ + 0.0001115*Q–0.0004108*λ^2 + 1.274e−07*λ*Q–3.597e−09*Q^2 + 7.149e−07*λ^3–4.124e−10*λ^4	0.8689
20	Mineral green	*f*_20_(*λ*,*Q*) = − 1.871 + 0.006661*λ + 0.001267*Q–5.503e−06*λ^2–3.215e−06*λ*Q-2.313e−08*Q^2 + 2.828e−11*λ*Q^2 + 2.281e−09*λ^2*Q	0.8487
21	Clam powder	*f*_21_(*λ*,*Q*) = − 7.013 + 0.04998*λ + 0.005879*Q–0.0001054*λ^2–1.448e−05*λ*Q–1.652e−07*Q^2 + 6.901e−08*λ^3 + 9.412e−09*λ^2*Q + 1.588e−10*λ*Q^2 + 2.669e−12*Q^3	0.8842
22	Verdigris	*f*_22_(*λ*,*Q*) = 24.57–0.08286*λ + 0.0003899*Q + 6.885e−05*λ^2–3.281e−07*λ*Q-3.717e−09*Q^2	0.8020
23	Carclazyte	*f*_23_(*λ*,*Q*) = 22.12–0.06904*λ + 0.0007074*Q + 5.301e−05*λ^2–5.374e−07*λ*Q − 1.492e−08*Q^2	0.8100

### Mathematical model for calculating the color damage of pigments

The color damage degree of pigment is related to three indexes, that is, exposure, SPD of the light source, and the relative response rate of pigment to exposure and SPD. The mathematical model of pigment color damage calculation in the visible light range can be defined as for the Eq. (3).

3 $$ D_{n} = \mathop \smallint \limits_{380}^{780} S\left( \lambda \right)f_{n} \left( {\lambda ,Q} \right)d\lambda $$

where *D*_*n*_ is the color damage degree of a certain pigment; *S*(*λ*) is the relative SPD of the irradiation light source, which can be measured by the spectrometer; *Q* is the amount of exposure with the unit of Wh/m^2^, which can be arbitrarily assigned; *f*_*n*_(*λ*,*Q*) is the relative responsivity function of a certain pigment to exposure and SPD, as shown in Table 2; n = 1 ~ 23, representing 1 ~ 23 kinds of pigments.

Taking graphite as an example, the function data of *f*_*8*_(*λ*,*Q*) in Table 2 was substituted into the Eq. (3), then the mathematical model for calculating the color damage of graphite can be obtained, as shown in the Eq. (4):

4 $$ D_{8} = \mathop \smallint \limits_{380}^{780} S\left( \lambda \right)\left( {3.608 - 0.01101*\lambda + 8.478e - 06*\lambda^{2} + 7.769e - 07*\lambda *Q - 1.551e - 09*Q^{2} - 5.851e - 12*\lambda *Q^{2} - 7.714e - 10*\lambda^{2} *Q} \right)d\lambda $$

According to the above method, the mathematical models *D*_1_ ~ *D*_23_ for calculating the color damage of all 23 pigments can be obtained.

## Discussion

**Figure 5:**
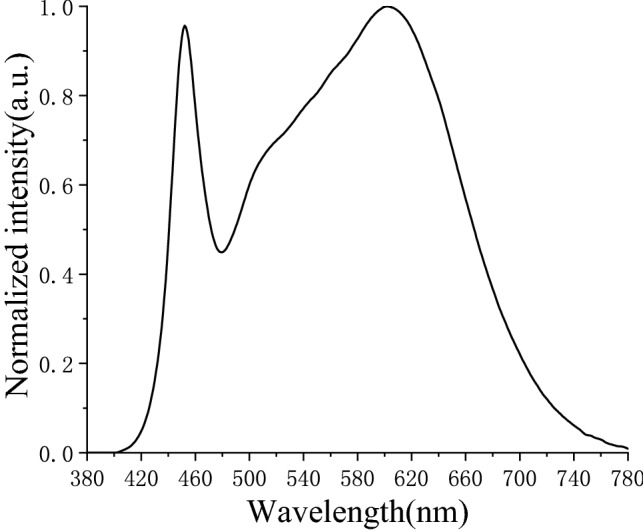
Relative SPD of experimental LED(4000 K).

**Figure 6:**
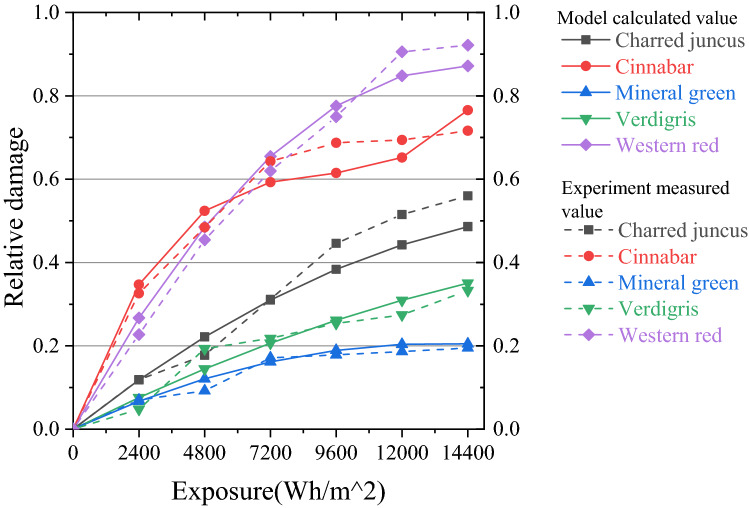
Comparison of experiment measured value and model calculated value.

To verify the accuracy of the mathematical model for color damage calculation, LED with color temperature of 4000 k was used as the experimental light source, whose SPD is shown in the Fig. 5. Five kinds of pigments, including charred juncus, cinnabar, mineral green, verdigris and western red, were irradiated with the exposure of 14,400 Wh/m^2^. Every 2400 Wh/m^2^ as a measurement cycle, the experimental measurement values of pigments’ color change were obtained. At the same time, we took the spectral power distribution *S*(*λ*) of the experimental light source and the damage responsivity function *f*_*n*_(*λ*, *Q*) of pigments into the Eq. (3) to obtain the color damage model calculated values of pigments. The comparison between the experiment measured values and the model calculated values (after normalization) is shown in Fig. 6.

Then, using paired t-test, the paired data of five pigments showed no statistical difference (P > 0.05) ^33^, as shown in Table 3. Therefore, the experiment measured values and the model calculated values are similarly matched, verifying the accuracy of the dyed artworks’ lighting damage model.

**Table 3:** Paired t-test analysis results.

Name	Mean ± SD		Difference (values measured—values calculated)	t	p
	Experiment measured values	Model calculated values			
Charred juncus	0.30 ± 0.21	0.28 ± 0.18	0.02	1.380	0.217
Cinnabar	0.51 ± 0.26	0.50 ± 0.25	0.01	0.435	0.679
Mineral green	0.13 ± 0.07	0.14 ± 0.08	−0.01	−1.591	0.163
Verdigris	0.19 ± 0.12	0.19 ± 0.13	−0.00	−0.385	0.713
Western red	0.55 ± 0.35	0.56 ± 0.33	−0.00	−0.221	0.833

## Conclusion

In this study, the mathematical models of multi-parameter coupling were established by using color difference of pigments in different narrow band lights and exposure combined with the method of data fitting. This method is feasible in researches on lighting damage.

In this study, the visual three-dimensional surface of 23 pigments’ color difference with wavelength and exposure was established, and the color damage law of 23 pigments under the coupling effect of light source SPD and exposure was obtained. Due to the difference characteristics of energy absorption and reflection of different materials, the damage law of different pigments is different. Take graphite as an example: firstly, the color damage increases with the increase of exposure, which is in line with the basic law of energy, that is, the accumulation of energy causes the accumulation of photochemical effect; secondly, the color damage increases with the decrease of wavelength, which is because black graphite absorbs the energy of all visible light bands, and with the decrease of wavelength, the spectral energy increasing leads to the increase of color damage.

The multi-parameter mathematical model $D_{n} = \mathop \smallint \limits_{380}^{780} S\left( \lambda \right) \cdot f_{n} \left( {\lambda ,Q} \right)d\lambda$ was established, and the accuracy of the model was verified by the experimental method of irradiating samples with typical light source. When the light source SPD and exposure *Q* are input, the color damage value of pigments can be obtained, which realizes the mathematical expression of the complex photochemical damage.
